# TWN-RENCOD: A novel method for protein binding site comparison

**DOI:** 10.1016/j.csbj.2022.12.014

**Published:** 2022-12-19

**Authors:** Kwang-Eun Choi, Anand Balupuri, Nam Sook Kang

**Affiliations:** Graduate School of New Drug Discovery and Development, Chungnam National University, 99 Daehak-ro, Yuseong-gu, Daejeon 34134, South Korea

**Keywords:** Protein binding site, Water network, Sunitinib, Binding site comparison method, Drug repurposing

## Abstract

Several diverse proteins possess similar binding sites. Protein binding site comparison provides valuable insights for the drug discovery and development. Binding site similarities are useful in understanding polypharmacology, identifying potential off-targets and repurposing of known drugs. Many binding site analysis and comparison methods are available today, however, these methods may not be adequate to explain variation in the activity of a drug or a small molecule against a number of similar proteins. Water molecules surrounding the protein surface contribute to structure and function of proteins. Water molecules form diverse types of hydrogen-bonded cyclic water-ring networks known as topological water networks (TWNs). Analysis of TWNs in binding site of proteins may improve understanding of the characteristics of binding sites. We propose TWN-based residue encoding (TWN-RENCOD), a novel binding site comparison method which compares the aqueous environment in binding sites of similar proteins. As compared to other existing methods, results obtained using our method correlated better with differences in wide range of activity of a known drug (Sunitinib) against nine different protein kinases (KIT, PDGFRA, VEGFR2, PHKG2, ITK, HPK1, MST3, PAK6 and CDK2).

## Graphical Abstract

**Figure:**
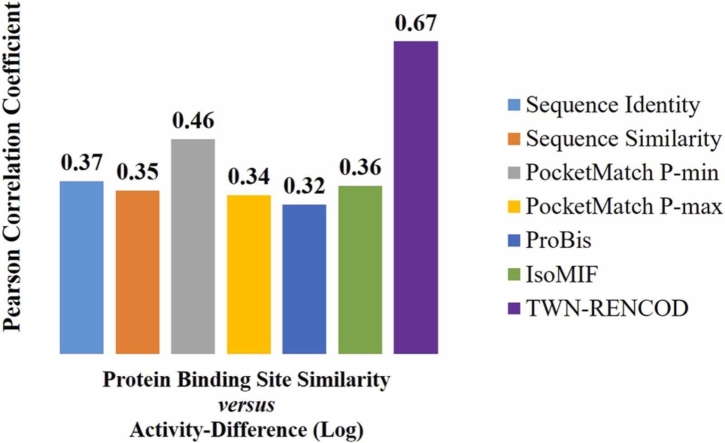

## 1 Introduction

The three-dimensional (3D) structure of proteins is of utmost importance to decipher the biological functions of proteins [1]. Analysis of the structures of proteins and particularly the investigation of binding sites, plays a key role in drug discovery [2]. Binding sites are closely related to protein function and understanding the characteristics of binding sites is a crucial step in structure based drug design [3]. Many different proteins possess similar binding sites and binding site comparisons provide useful suggestions for the repurposing of known drugs, the identification of possible off-targets and the establishment of polypharmacology [4], [5].

A number of computational techniques are available to exploit protein structures for addressing various scientific problems [6], [7]. Several computational methods are developed to compare ligand binding sites of available protein structures. Binding site comparison methods can be majorly classified into three categories namely residue-, surface- and interaction-based methods. These methods are described in detail elsewhere [8]. Briefly, residue-, surface- and interaction-based methods often make use of structural descriptors, graphs and molecular interaction fields, respectively. The number of binding site comparison methods is still growing and it is becoming more and more difficult to select the most appropriate method for a specific area of research [9].

In this study, we applied various binding site comparison methods to X-ray crystal structures of multiple proteins bound to the same ligand, to explore if binding site similarity can explain difference in activity of that ligand against different proteins. Sunitinib is a small-molecule, multi-targeted kinase inhibitor that was approved by the U.S. Food and Drug Administration (FDA) for the treatment of renal cell carcinoma and imatinib-resistant gastrointestinal stromal tumor [10]. Sunitinib exhibits a broad range of inhibitory activity against various kinases [11]. The X-ray crystal structures of a significant number of protein kinases co-crystallized with Sunitinib can be found in the Protein Data Bank (PDB). Staurosporine is another potent multi-targeted kinase inhibitor with more crystal structures in the PDB. However, it demonstrates a narrow inhibitory activity range against different kinases. Accordingly, we selected Sunitinib bound crystal structures of various kinases in similar conformation for this study.

We compared binding site of nine protein kinases namely tyrosine-protein kinase Kit (KIT), Tyrosine-protein kinase ITK/TSK (ITK), cyclin-dependent kinase 2 (CDK2), vascular endothelial growth factor receptor (VEGFR2), p21-activated kinase (PAK6), mammalian STE20-like protein kinase 3 (MST3), platelet-derived growth factor receptor A (PDGFRA), hematopoietic progenitor kinase 1 (HPK1) and phosphorylase kinase subunit gamma-2 (PHKG2) using different binding site comparison methods. We selected PocketMatch [12], ProBiS [13] and IsoMIF [14] programs as the representatives for residue-, surface- and interaction-based methods because standalone versions of these software are freely available for non-commercial use and these methods exhibit high sensitivity and high specificity [8]. Comparison of binding sites of different protein kinases co-crystallized with Sunitinib indicated that existing methods may not be adequate to explain the difference in activity of this drug against analyzed proteins. This may be due to the reason that most of the currently available binding site comparison methods do not focus much on the local aqueous environment in the binding sites.

Water molecules surrounding the protein surface are fundamental in mediating protein folding, structure, function and ligand binding [15]. Proteins may also effect the organization of water molecules and thus probing water molecules in the binding site of proteins may pave a way to further understand the characteristics of binding sites [16]. Hydrogen-bonded networks are often formed by water molecules in the binding site of proteins. We are actively conducting research on the water network analysis for the past several years. Earlier, we proposed topological water network (TWN) analysis method to determine the water networks without explicit free energy calculations [17], [18], [19], [20], [21], [22], [23], [24]. We successfully employed this method to design kinase inhibitors [20], [21], to describe kinase selectivity [19], to examine protein–ligand binding [20], to investigate drug repurposing [22], to probe protein hydration [23] and to understand protein folding [24].

Here, we introduce TWN-based residue encoding (TWN-RENCOD), a novel binding site comparison method. Unlike our previous TWN works [17], [18], [19], [20], [21], [22], [23], [24], TWN-RENCOD considers distances between TWNs and binding site residues to compute similarity between the binding sites of proteins. We investigated binding sites of different protein kinases co-crystallized with Sunitinib using our method and found that differences in the inhibitory activity of Sunitinib against analyzed kinases correlated better with TWN-RENCOD values.

## 2 Material and methods

### 2.1 Workflow

**Fig. 1:**
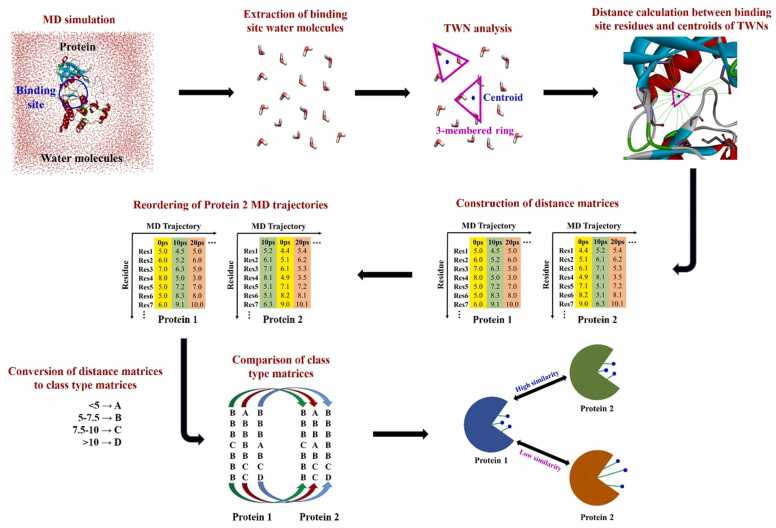
The work flow for TWN-RENCOD methodology.

In the TWN-RENCOD method (Fig. 1), initially molecular dynamics (MD) simulation is performed on the apo state of the protein. Protein is restrained and only water molecules are allowed to move during MD simulation. Next, 3-membered ring TWNs are analyzed in all MD trajectories. Then, a distance matrix is constructed by computing the distances between centroid of each 3-membered ring TWN and backbone atoms of each residue within the binding site of the protein. Similarly, another distance matrix is constructed for the protein to be compared. Since TWNs in the same site may be observed in different MD trajectories of the compared proteins, MD trajectories of the second protein are reordered to match TWNs of the first protein based on the minimum difference in distance values of each trajectory. In the reordering process (Fig. S1), all MD trajectories of the second protein are compared with each MD trajectory of the first protein based on the distances values. For example, all trajectories of the second protein (0 ps, 10 ps, 20 ps, 30 ps, 40 ps, 50 ps, 60 ps, 70 ps, 80 ps, etc.) are compared with the first trajectory (0 ps) of the first protein. If difference-in-distance is minimum for 30 ps trajectory of the second protein, then this trajectory is reordered as 0 ps trajectory for the second protein. Next, remaining trajectories (0 ps, 10 ps, 20 ps, 40 ps, 50 ps, 60 ps, 70 ps, 80 ps, etc.) of the second protein are compared with the second trajectory (10 ps) of the first protein. If difference-in-distance is minimum for 70 ps trajectory of the second protein, then this trajectory would be reordered as 10 ps trajectory for the second protein. Similarly, other remaining trajectories (0 ps, 10 ps, 20 ps, 40 ps, 50 ps, 60 ps, 80 ps, etc.) of the second protein are compared with remaining trajectories (20 ps, 30 ps, 40 ps, 50 ps, 60 ps, 70 ps, 80 ps, etc.) of the first protein. Afterwards, reordered distance matrix is created for the second protein. The reordered distance matrix of the second protein as well as distance matrix of the first protein are converted into class type matrices based on the distance range (class A:<5 Å, class B: 5–7.5 Å, class C: 7.5–10 Å and class D:>10 Å). The distance classes are defined according to the previous studies which described various hydration layers around proteins [25], [26], [27]. Finally, class type matrices of first and second proteins are compared to compute similarity between proteins. The similarity is calculated as ratio of matched class pairs to the total number of class pairs.

### 2.2 Protein preparation

Sunitinib bound X-ray crystal structures of nine human protein kinases namely KIT (PDB: 3G0E) [28], PDGFRA (PDB: 6JOK), VEGFR2 (PDB: 4AGD) [29], PHKG2 (PDB: 2Y7J), ITK (PDB: 3MIY) [30], HPK1 (PDB: 6NFY) [31], MST3 (PDB: 4QMZ) [32], PAK6 (PDB: 4KS8) [33] and CDK2 (PDB: 3TI1) [34] were obtained from the RCSB PDB. All non-protein molecules including co-crystallized Sunitinib were removed from PDBs and protein structures were further processed with the ‘Prepare Protein’ module of Discovery Studio 2022 (BIOVIA, San Diego, CA, USA). During protein preparation, missing residues and hydrogen atoms were added, and bond orders and formal charges were assigned. Protonation states were assigned at pH 7.4.

### 2.3 MD simulation

All MD simulations were performed using GROMACS package, version 5.1.4 [35] and CHARMM27 all-atom force field [36]. The protein molecule was enclosed in a cubic box and solvated by TIP3P water molecules [37] with a margin distance of 15 Å. System was neutralized by adding appropriate number of counter ions and energy minimized with the steepest descent algorithm up to 50,000 steps. System was equilibrated in the NVT (constant number of particles, volume and temperature) ensemble for 100 ps using V-rescale thermostat [38]. This was followed by equilibration in the NPT (constant number of particles, pressure and temperature) ensemble for 200 ps using Parrinello-Rahman barostat [39]. Finally, production run was executed at 298 K and 1 bar pressure for 10 ns. Protein was restrained during the production run. MD simulations were performed under periodic boundary conditions. Long-range electrostatics were computed using the Particle mesh Ewald method [40] and short-range electrostatics were truncated at 12 Å. All bond lengths were constrained using the linear constraint solver algorithm [41]. MD trajectories were recorded every 1 ps for the analysis. For each protein, 10 ns MD simulation was performed and 1000 frames were recorded.

### 2.4 TWN analysis

**Fig. 2:**
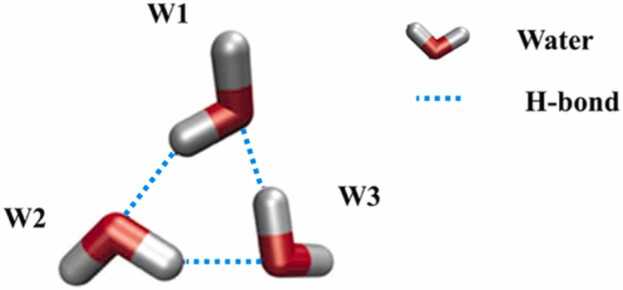
An illustration of a 3-membered ring TWN. Water molecules involved in the 3-membered ring TWN are represented by stick models (W1-W3) while hydrogen bond interactions are represented by dotted lines.

Water molecules form a number of hydrogen-bonded cyclic water-ring networks recognized as TWNs [17], [18], [19], [20]. The 3-membered ring TWN is a type of TWNs that includes three water molecules forming a hydrogen-bonded cyclic water-ring network (Fig. 2).

Potential functions considered in TWNs involve a rigid TIP3P water model. The Lennard-Jones and Coulomb potentials are used to model the interactions between water molecules. The interaction potential energy between water molecules is estimated according to the equation below.

$$v\left(a,b\right)=\sum_{i}^{\mathrm{on}a}\sum_{j}^{\mathrm{on}b}\frac{q_{i}q_{j}e^{2}}{r_{\mathrm{ij}}}+\frac{A}{{r_{\mathrm{oo}}}^{12}}-\frac{C}{{r_{\mathrm{oo}}}^{6}}$$

where:

v(*a*, *b*) = interaction potential energy between water molecules *a* and *b*
r_oo_ = distance between oxygen atoms
q_i_ = partial charge on the *i* site (−0.834*e*)
q_j_ = partial charge on the *j* site (0.417*e*)
r_ij_ = distance between *q*_*i*_ and *q*_*j*_
*A* = repulsive force between *i* and *j* (582,000 kcal∙Å^12∙^mol^−1^)
*C* = attractive force between *i* and *j* (595 kcal∙Å^6∙^mol^−1^)

To determine hydrogen bond between water molecules, energy criterion of − 2.25 kcal mol^−1^ is considered. Comprehensive details of TWN method are provided in our previous publications [17], [18], [19], [20], [21], [22], [23], [24]. For TWN analysis, MD simulation was performed on each protein in the apo state and all MD trajectories were extracted. The 3-membered ring TWNs were analyzed in each extracted MD trajectory according to the equation described above.

### 2.5 Binding site selection

The kinase–ligand interaction fingerprints and structure (KLIFS) database contains a consistent alignment of 85 kinase ligand binding site residues [42]. All structures of catalytic domains of protein kinases deposited in the PDB have been processed in a consistent manner by systematically analyzing the structural features and molecular interaction fingerprints of a predefined set of 85 binding site residues with bound ligands. The binding pocket composition feature allows the selection of specific amino acids at specific residue positions in the binding pockets of various kinases. In our study, binding site of each protein kinase was defined based on the co-crystallized ligand (Sunitinib) in corresponding PDB structures. The residues within 6 Å of the co-crystallized ligand were considered as the binding site residues. As the number of residues within the specified range slightly varied from one kinase to another, the maximum number of residues within that range for any kinase was taken into account to select the same number of binding site residues for each kinase. We referred KLIFS database for selecting the corresponding binding site residues in all kinases (Fig. S2). As the corresponding binding site residues may differ from one kinase to another due to dissimilar side chains, we considered only the backbone atoms (N, Cα, C and O) of each residue and not the side chain atoms in the TWN-RENCOD method. A total of 16 binding site residues and 64 atoms were included in TWN-RENCOD calculations for each protein.

### 2.6 TWN-RENCOD

The 3-membered ring TWNs were firstly analyzed within the binding site of a protein in all the MD trajectories. Then, distances between backbone atoms (N, Cα, C and O) of each residue within the binding site of protein and centroid of each 3-membered ring TWN were calculated. A distance matrix was created based on the calculated distances. This matrix was further converted into a class type matrix according to the distance range (class A:<5 Å, class B: 5–7.5 Å, class C: 7.5–10 Å and class D:>10 Å). Same procedure was followed for creating distance matrix for the other protein. As similar TWNs in a particular location of the binding sites of proteins existed in different MD trajectories of proteins, MD trajectories of the second protein were reordered according to MD trajectories of the first protein. Reordering was done depending on the minimum difference in the distance values of each MD trajectory of both proteins. Afterwards, reordered distance matrix of the second protein was also converted into the class type matrix and compared with class type matrix of the first protein to calculate similarity between proteins. Similarity was defined as the ratio of matched class pairs to the total number of class pairs.

### 2.7 Binding site sequence identity and similarity

Binding site sequence identity and similarity were calculated with the ‘Align Sequences’ module of Discovery Studio 2022 (BIOVIA, San Diego, CA, USA). Sequence identity and similarity percentage values were converted into decimal values. The ‘Align Sequences’ module aligns two or more protein sequences using the Align123 algorithm, a progressive pairwise alignment algorithm modified from the CLUSTAL W program [43]. For each pair of protein kinases, binding site residues’ sequences were aligned to determine binding site sequence identity and similarity (Fig. S3).

Additionally, binding site similarity was calculated using three other programs namely PocketMatch [12], ProBiS [13] and IsoMIF [14] which represent residue-, surface- and interaction-based methods [8]. PocketMatch (version 2) calculates binding-site similarity according to structural descriptors such as residue nature and interatomic distances. It computes all against all-atom distance pairs in the two binding sites and compares sorted lists of distances to determine similarity. It provides P-max and P-min scores which represent the ratio of matched distance pairs to the total number of distance pairs in the longer and shorter binding site, respectively. ProBiS aligns and superimposes protein binding sites, and calculates similarity by comparing solvent accessible surface over the protein atoms. It provides Z-scores which were converted into percentile and then to decimal values. IsoMIF calculates similarity on the basis of molecular interaction fields which describe the putative interactions of binding site residues with ligand atoms. The similarity score is computed as Tanimoto similarity between fields.

### 2.8 Estimation of binding free energy

Binding energy of Sunitinib against each protein kinase was estimated with ‘Calculate Binding Energies’ module of Discovery Studio 2022 (BIOVIA, San Diego, CA, USA) using the CHARMm forcefield. This module calculates binding energy according to the equation below:

Energy_Binding_ = Energy_Complex_ - Energy_Ligand_ - Energy_Protein_

## 3 Results and discussion

Sunitinib exhibits a broad range of inhibitory activity against nine representative protein kinases (Table 1).

**Table 1:** Inhibitory activity of Sunitinib against various protein kinases [11].

Protein	Abbreviation	PDB-ID	K_d_ (nM)
Tyrosine-protein kinase Kit	KIT	3G0E	0.37
Platelet-derived growth factor receptor A	PDGFRA	6JOK	0.79
Vascular endothelial growth factor receptor	VEGFR2	4AGD	1.50
Phosphorylase kinase subunit gamma-2	PHKG2	2Y7J	5.90
Tyrosine-protein kinase ITK/TSK	ITK	3MIY	13.00
Hematopoietic progenitor kinase 1	HPK1	6NFY	16.00
Mammalian STE20-like protein kinase 3	MST3	4QMZ	63.00
p21-activated kinase	PAK6	4KS8	2400.00
Cyclin-dependent kinase 2	CDK2	3TI1	> 10,000.00

**Fig. 3:**
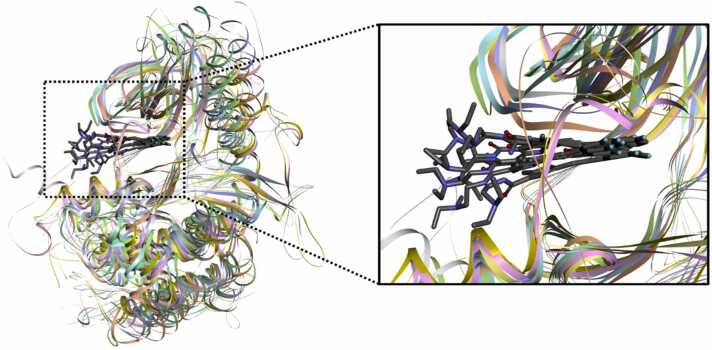
Superimposed crystal structures of Sunitinib (gray) bound KIT (pink), PDGFRA (yellow), VEGFR2 (chartreuse), PHKG2 (cyan), ITK (blue), HPK1 (green), MST3 (gray), PAK6 (purple) and CDK2 (orange).

The X-ray crystal structures of these protein kinases co-crystallized with Sunitinib are available in the RCSB PDB [28], [29], [30], [31], [32], [33], [34]. As displayed in Fig. 3, these protein structures exist in the similar conformation and Sunitinib exhibits similar binding mode against proteins. The binding mode shows that oxoindole moiety of Sunitinib occupies ATP-binding hinge region while diethylamino moiety points toward the solvent exposed region. Oxoindole and pyrrole moieties display a similar planar conformation, however, diethylamino moiety is slightly disordered.

**Fig. 4:**
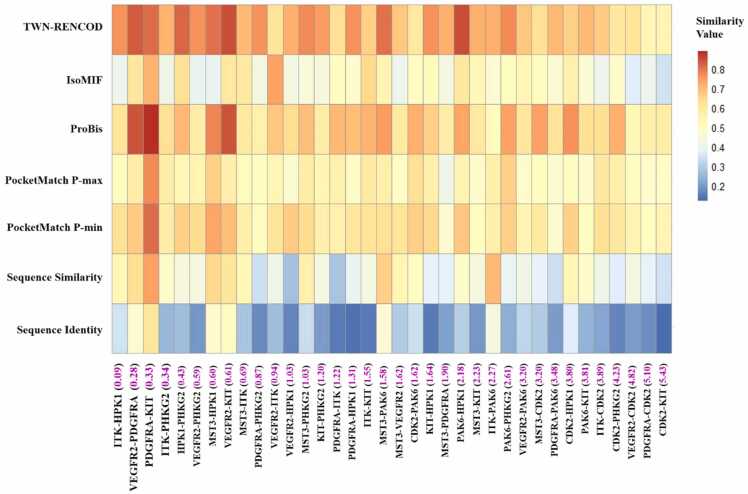
A heatmap of the binding site similarity values for various protein-pairs computed using different methods, blue colors indicate low similarity values and red colors indicate high similarity values. The differences in activity (log) of Sunitinib for various protein-pairs are shown in parentheses in the magenta color.

**Fig. 5:**
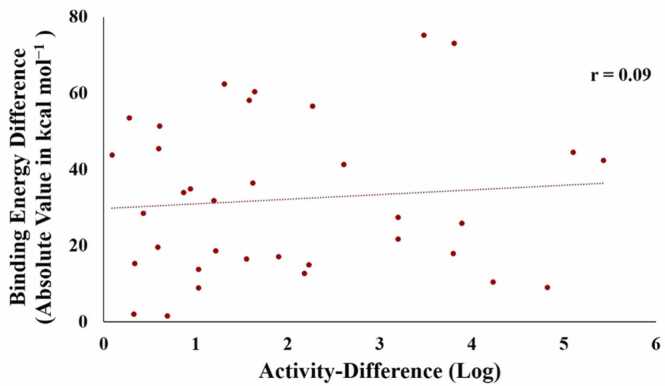
Correlation between the differences in activity (log) and the differences in binding energy (absolute values in kcal mol^−1^) of Sunitinib for various protein-pairs.

Binding sites of kinases were compared to understand the multi-target activity of Sunitinib. Firstly, we calculated binding site sequence identity and similarity values between proteins (Fig. 4 and Table S1), and studied the relationship between these values and differences in activities of Sunitinib (Table 2). A low correlation with a Pearson correlation coefficient (r) of 0.37 was observed between activity-difference (log) and sequence identity values. Similarly, a low correlation (r = 0.35) was observed with sequence similarity values. Consequently, we estimated the binding energy of Sunitinib against proteins (Table S2) and inspected the correlation between binding energy difference and activity-difference (log) values (Fig. 5). Unexpectedly, we found a poor correlation (r = 0.09) between them.

**Table 2:** Correlation between the differences in activity (log) of Sunitinib and the binding site similarity values for various protein-pairs.

Protein binding site similarity assessment	Correlation coefficient		
	Pearson	Kendall	Spearman
Sequence Identity	0.37	0.25	0.34
Sequence Similarity	0.35	0.29	0.40
PocketMatch P-min	0.46	0.32	0.47
PocketMatch P-max	0.34	0.24	0.35
Pro-Bis	0.32	0.15	0.26
Iso-MIF	0.36	0.15	0.22
TWN-RENCOD	0.67	0.47	0.62

We decided to compare binding sites using other available methods. A number of protein binding site comparison methods are available today, which can be classified into three categories namely residue-, surface- and interaction-based methods [8]. We chose one representative method from each category for further exploration. Due to the availability of free standalone versions as well as high sensitivity and high specificity [8], PocketMatch, ProBiS and IsoMIF programs were selected. We calculated binding site similarity between proteins using these methods (Fig. 4 and Table S1) and examined the relationship between various similarity values and activity-difference (log) values (Table 2). Similar to binding site sequence identity and similarity, low correlations were obtained with PocketMatch P-max (r = 0.34), ProBiS (r = 0.32) and IsoMIF (r = 0.36) similarity values. PocketMatch P-min score (r = 0.46) showed slightly better correlation, but not much. PocketMatch, ProBiS and IsoMIF methods as well as other existing binding site comparison methods do not focus much on the local aqueous environment around binding sites. This may contribute to the low correlation between activity-difference (log) and similarity values.

The aqueous environment is a pervasive factor which determines the structure, function and dynamics of proteins. Furthermore, water molecules within binding site of a protein play a crucial role in mediating the protein-ligand interactions. Proteins may also influence the organization of water molecules around the protein surface. Investigation of binding site water molecules can provide valuable information about the binding site characteristics. Water molecules often form hydrogen-bonding networks in the protein binding sites. Our research group previously developed an algorithm known as TWN to analyze water networks [17], [18], [19], [20]. We successfully used TWN to address various scientific problems [17], [18], [19], [20], [21], [22], [23], [24]. Here, we have developed a novel method based on TWN algorithm to compare protein binding sites and named it as TWN-RENCOD. We calculated binding site similarity between proteins using our method and examined the relationship between TWN-RENCOD similarity and activity-difference (log) values. Interestingly, a high correlation (r = 0.67) was obtained between them (Table 2). With the highest correlation coefficient, the proposed method outperformed existing methods in explaining the variation in the activity of Sunitinib against several protein kinases.

In addition to the Pearson's correlation, we computed Kendall and Spearman correlation coefficients using R software (R Core Team 2020, A language and environment for statistical computing, R Foundation for Statistical Computing, Vienna, Austria, URL http://www.r-project.org/). Kendall and Spearman computations also showed similar performance as demonstrated by Pearson's computation (Table 2). Furthermore, Pearson correlation coefficients were compared with each other using the cocor package [44]. All tests were one-tailed, and p-values were used to determine statistical significance (Table S3). A p-value less than 0.05 is considered statistically significant. TWN-RENCOD correlations seem to be statistically significant as most of the p-values are within the acceptance range (<0.05). Whereas, correlations using other methods seem to be comparatively insignificant (p-value>0.05).

The present study highlights the importance of water molecules in protein binding site and suggests that existing methods are insufficient to compare protein binding sites. Water network based comparison of protein binding sites can provide new insights about the characteristics of binding sites. Thus, the proposed method can be used to compare binding sites of similar proteins for the drug repurposing and for the development of new molecules for target proteins. However, it should be noted that unlike PocketMatch, ProBiS and IsoMIF programs, TWN-RENCOD is not suitable for the analysis of large number of proteins as this method involves MD simulations. Furthermore, TWN-RENCOD is developed to compare binding sites of similar proteins.

## 4 Conclusions

We present TWN-RENCOD, a new method to compare protein binding sites. The proposed method computes binding site similarity based on water networks observed in binding sites of proteins. As compared to various existing protein binding site comparison methods, TWN-RENCOD showed much better performance in explaining the variation in activity of Sunitinib against several protein kinases. Outcomes of this study suggest that comparison of protein binding sites using TWN-RENCOD can provide novel insights about the characteristics of binding sites which can be exploited for drug discovery and development.

## Funding

This research was funded by the Basic Science Research Program through the 10.13039/501100003725National Research Foundation of Korea (NRF) funded by the Ministry of Science, ICT, and Future Planning (NRF2020R1A2C100691511).(NRF2020R1A2C100691511).

## Declaration of Competing Interest

The authors declare that they have no known competing financial interests or personal relationships that could have appeared to influence the work reported in this paper.
