# Supplementary material for: TWN-RENCOD: A novel method for protein binding site comparison
Source: Comput Struct Biotechnol J. 2022 Dec 19;21:425–31. doi: 10.1016/j.csbj.2022.12.014 (PMC9798139; doi:10.1016/j.csbj.2022.12.014)

**Fig. S1.** Reordering process of MD trajectories in TWN-RENCOD method. Residue backbone atoms are represented as N, Cα, C and O while centroid of 3-membered ring TWN is represented as R3C.

**
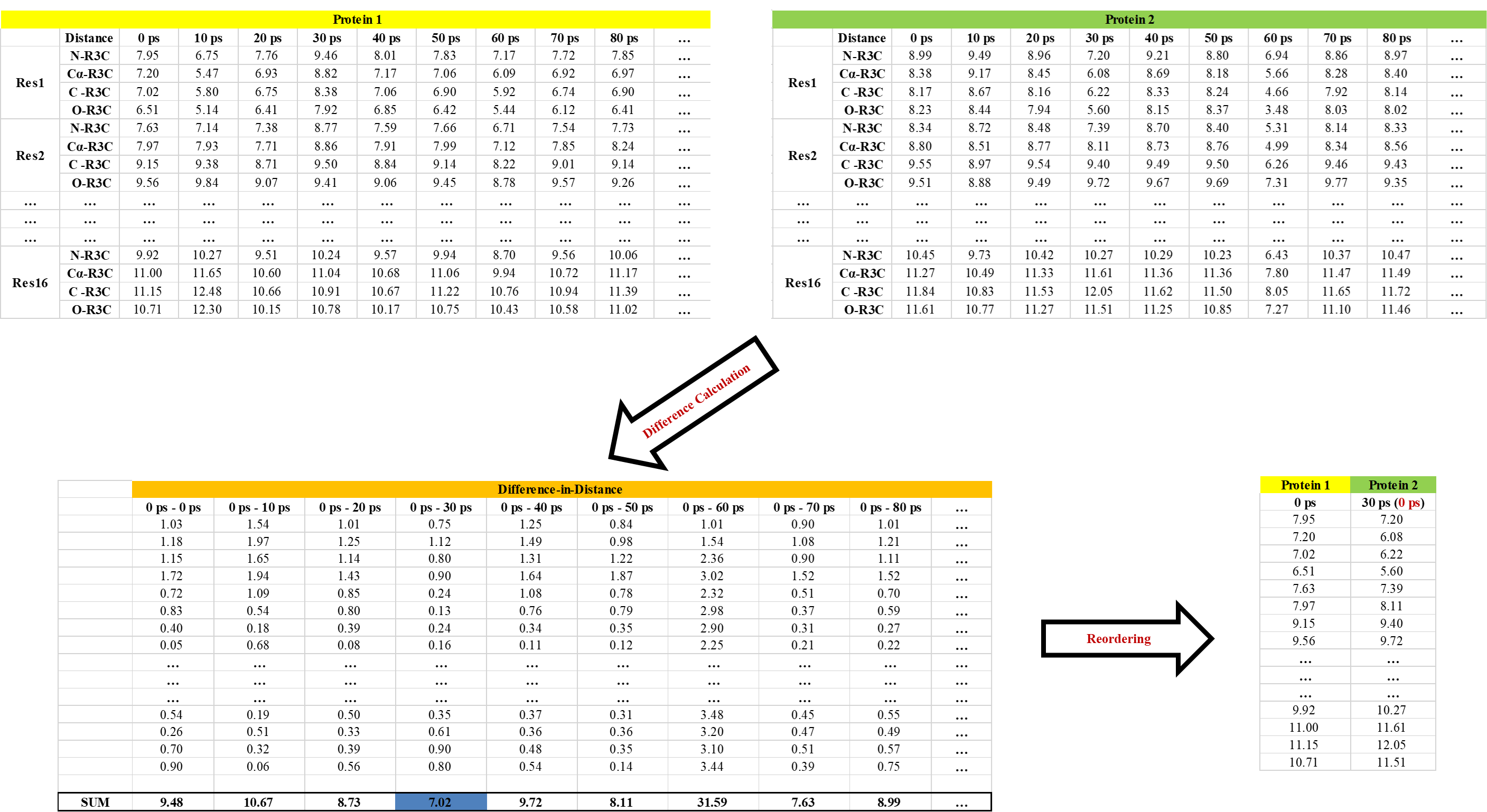
**

**
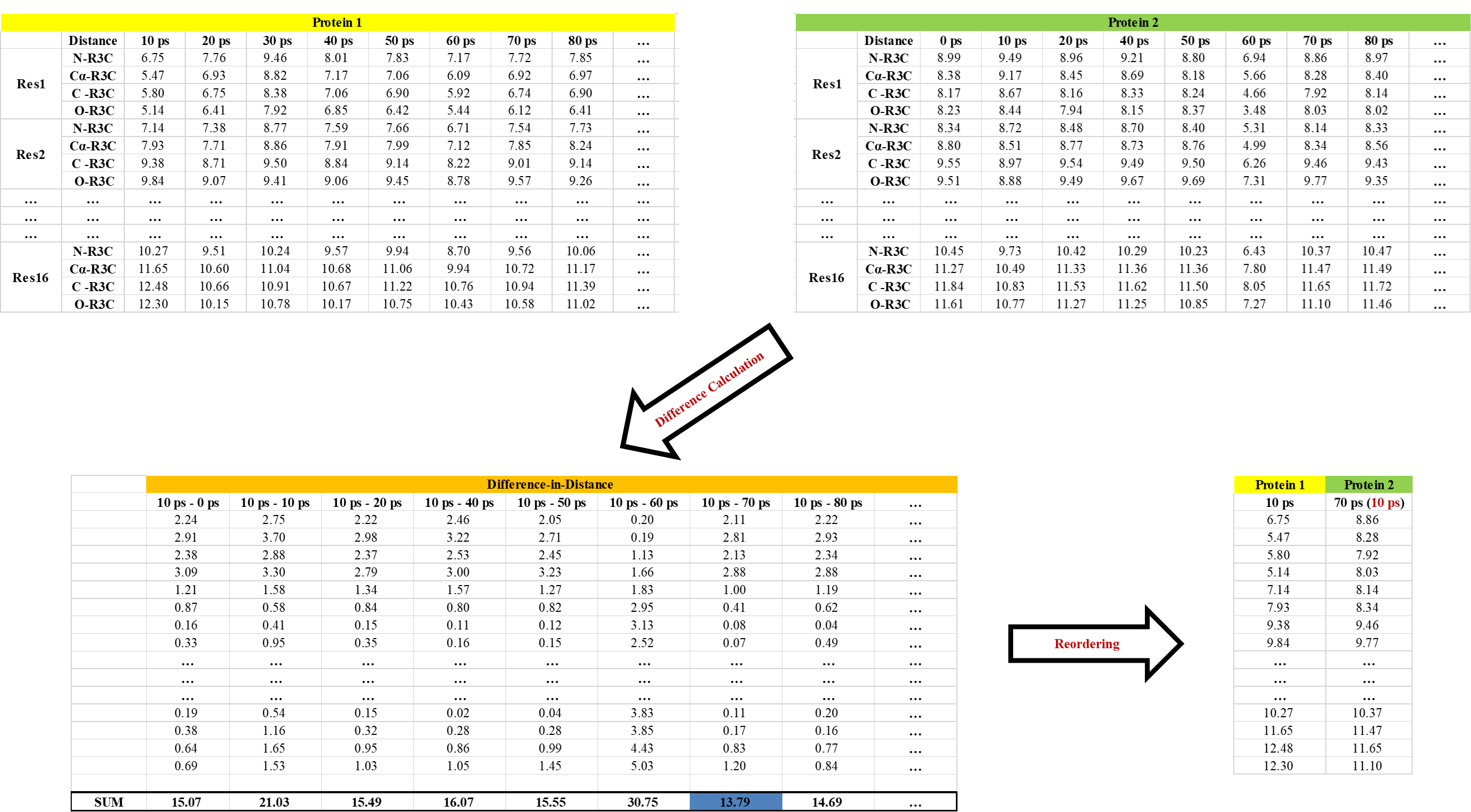
**

**Fig. S2.** Catalytic site information from KLIFS database. Binding site residues of each kinase are highlighted by red boxes.


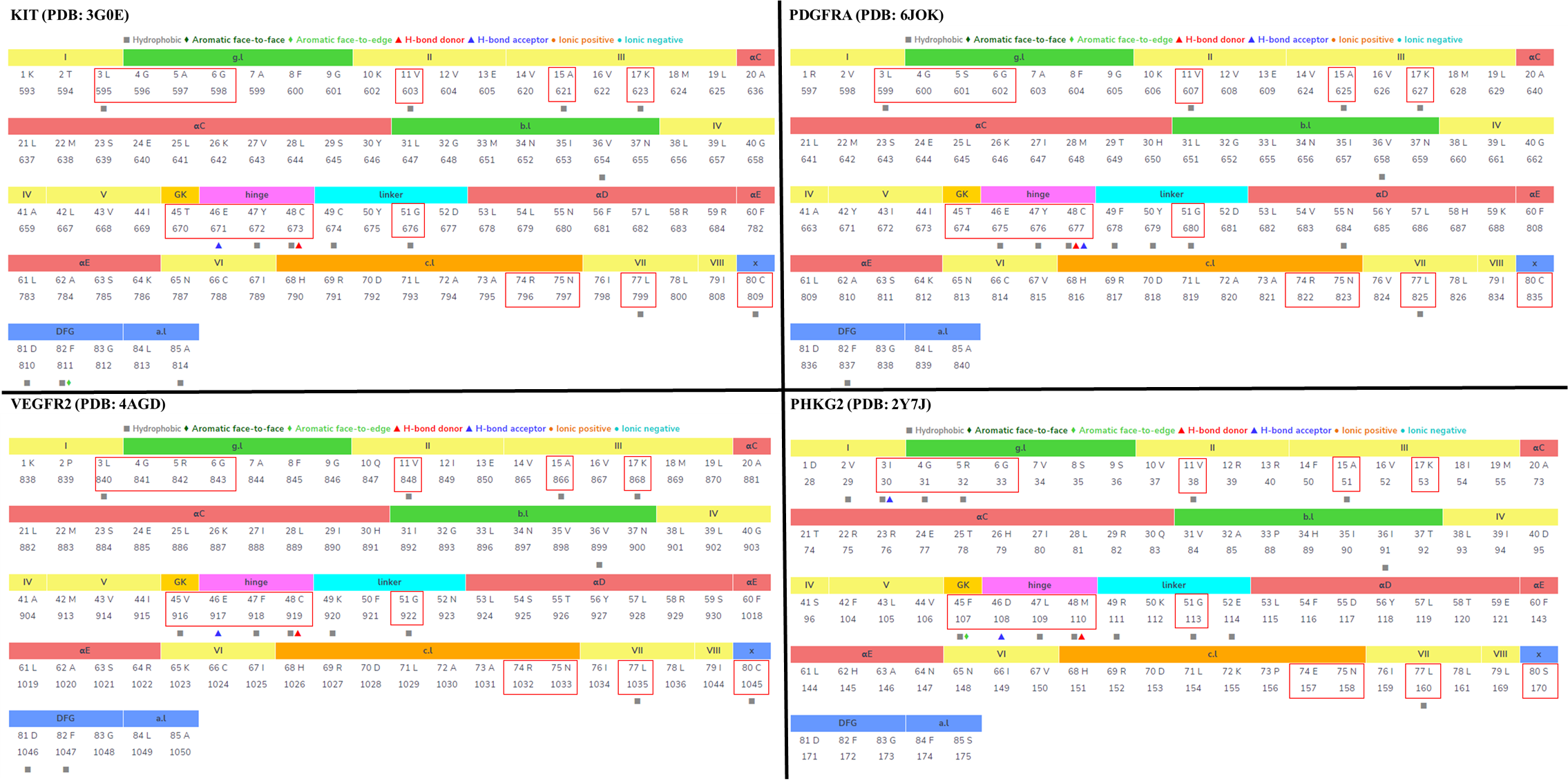


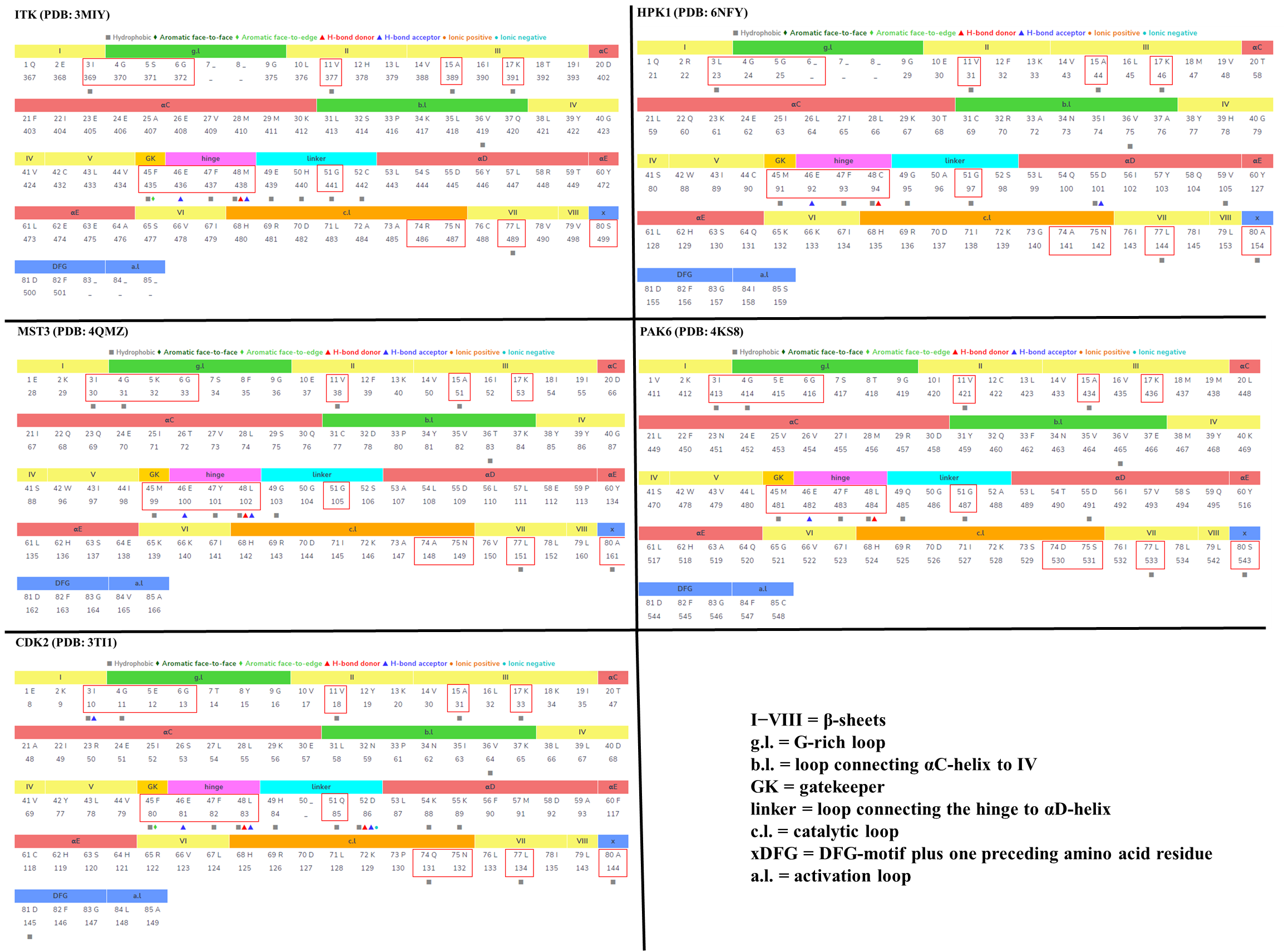


**Fig. S3.** The binding site sequence alignment for various protein-pairs.


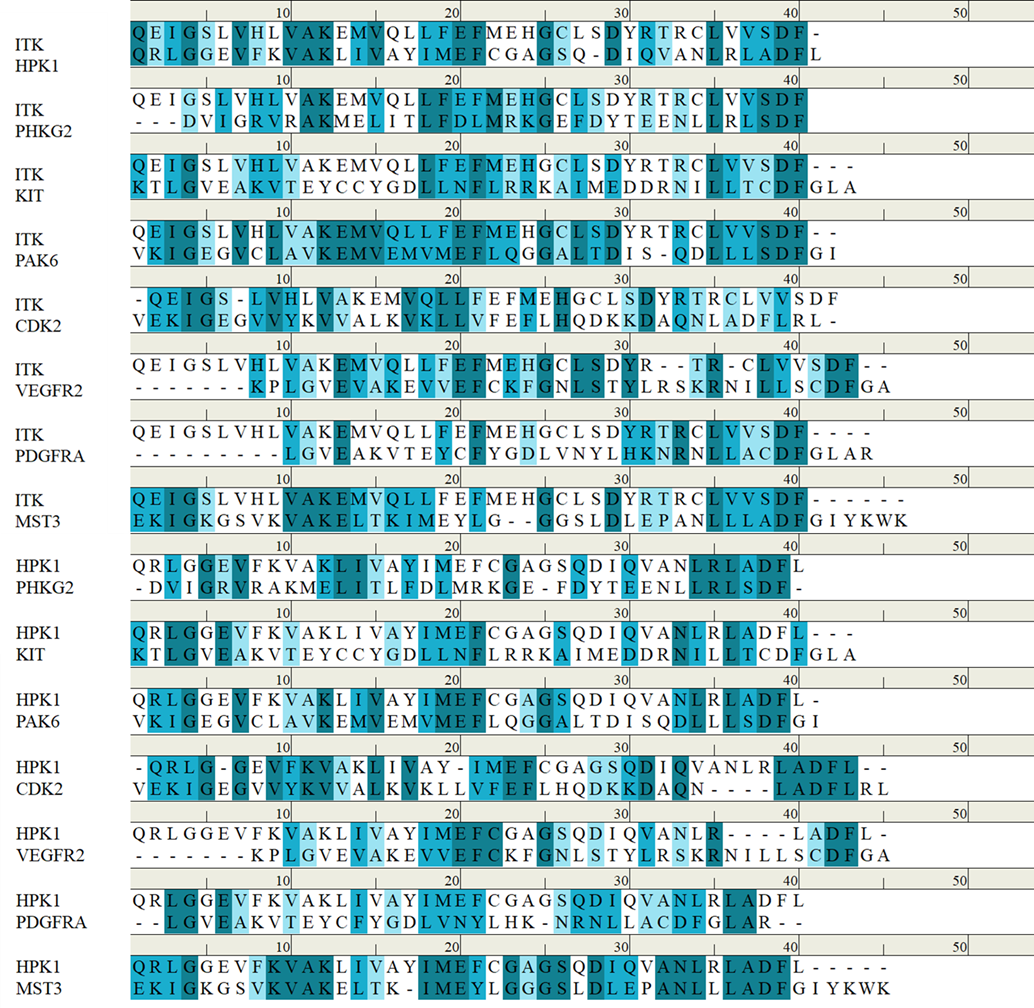


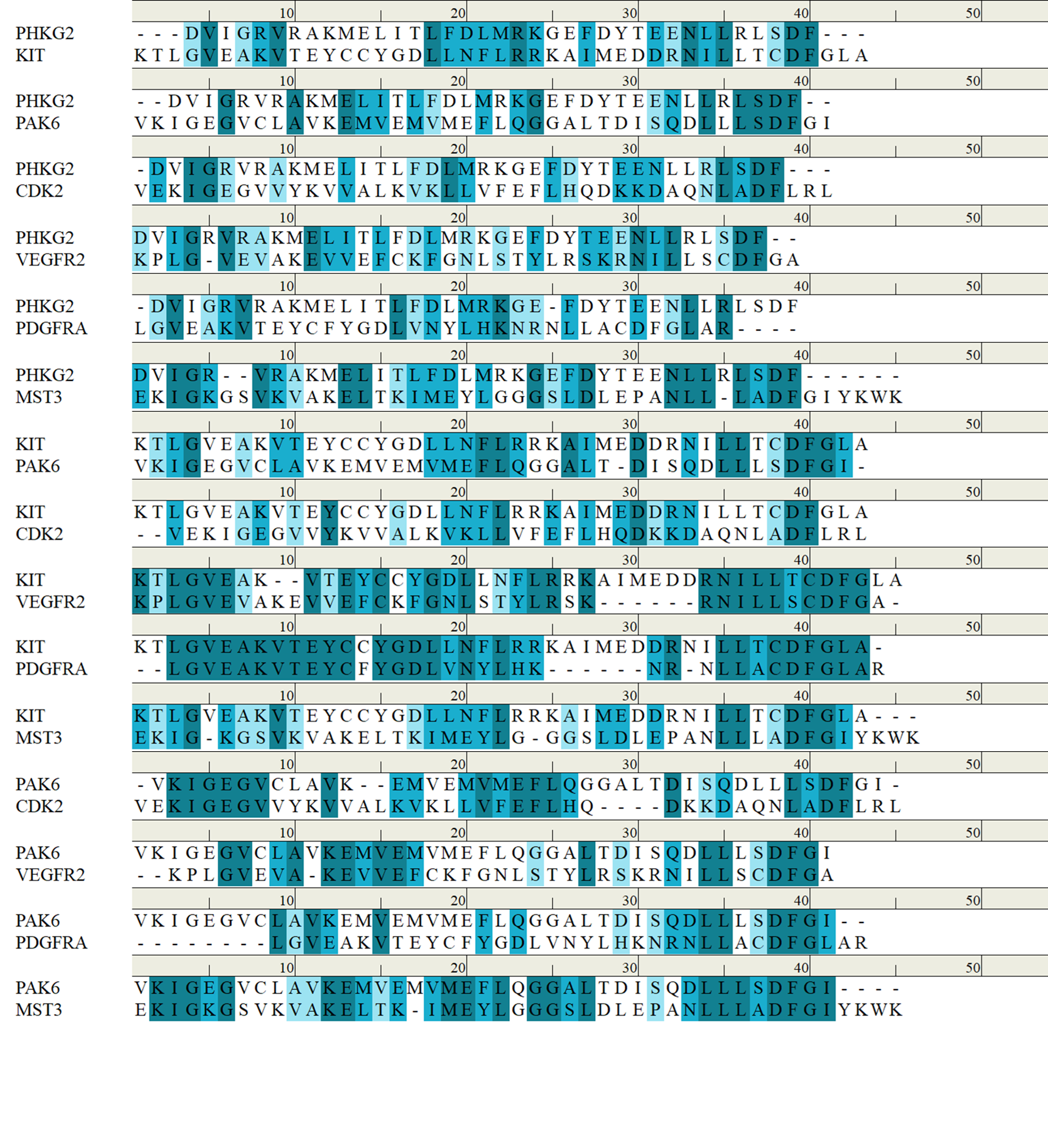


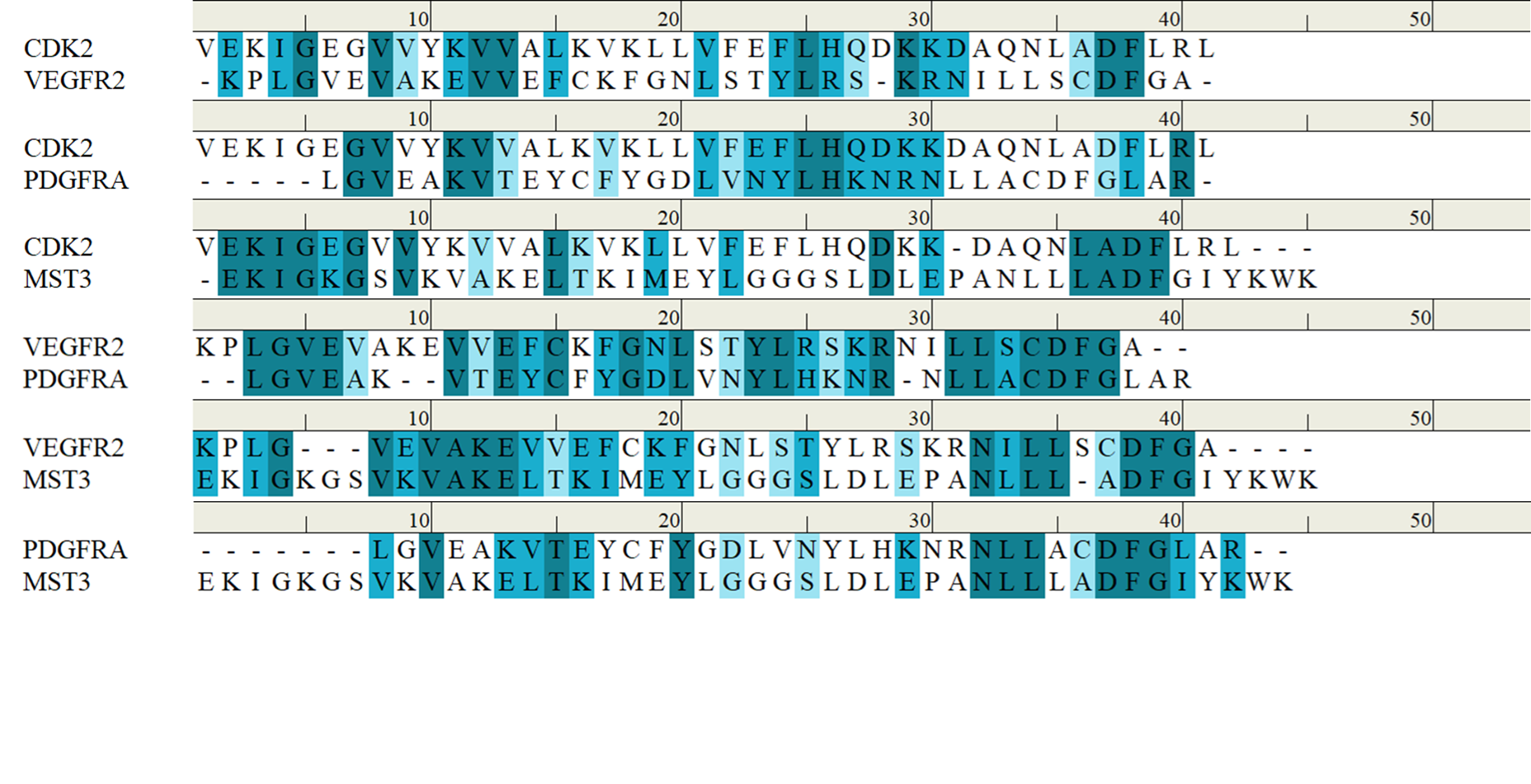

Supplement: Supplementary file 2 — Supplementary material [file mmc2.docx]
